# Group-housed females promote production of asexual ootheca in American cockroaches

**DOI:** 10.1186/s40851-017-0063-x

**Published:** 2017-03-13

**Authors:** Ko Katoh, Masazumi Iwasaki, Shouhei Hosono, Atsushi Yoritsune, Masanori Ochiai, Makoto Mizunami, Hiroshi Nishino

**Affiliations:** 1 Graduate School of Life Science, Hokkaido University, Sapporo, 060-0810 Japan; 2 Research Institute for Electronic Science, Hokkaido University, Sapporo, 060-0812 Japan; 3 The Institute of Low Temperature Science, Hokkaido University, Sapporo, 060-0819 Japan; 4 Faculty of Science, Hokkaido University, Sapporo, 060-0810 Japan; 5 Present address: Division of Applied Entomology and Zoology, Graduate School of Agriculture, Tamagawa University, Machida, Tokyo, Japan

**Keywords:** Cockroaches, Sexual reproduction, Parthenogenesis, group effect, Sex pheromone, Antenna, Chemosensory signal, Periplanone

## Abstract

**Background:**

Facultative parthenogenesis, seen in many animal phyla, is a reproductive strategy in which females are able to generate offspring when mating partners are unavailable. In some subsocial and eusocial insects, parthenogenesis is often more prevalent than sexual reproduction. However, little is known about how social cooperation is linked to the promotion of parthenogenesis. The domiciliary cockroach *Periplaneta americana* is well-suited to addressing this issue as this species belongs to the superfamily Blattoidea, which diverged into eusocial termites and shows facultative parthenogenesis.

**Results:**

We studied environmental factors that influence asexual production of ootheca using behavioral assays in *P. americana*. When more than three virgin females immediately after the imaginal molt were kept together in a small sealed container, they tended to produce egg cases (oothecae) via parthenogenesis earlier than did isolated females, resulting in apparent synchronization of ootheca production, even among females housed in different containers. In contrast, virgin females housed with genitalia-ablated males or group-housed females with antennae ablated did not significantly promote ootheca production compared to isolated females. Daily addition of the primary sex pheromone component to the container did not promote ootheca production in isolated females. Another line of study showed that grouped females make parthenogenesis more sustainable than previously known; a founder colony of 15 virgin females was sufficient to produce female progeny for a period of more than three years.

**Conclusions:**

Group-housed females promote and stabilize asexual ootheca production compared to isolated females, and that this promotion is triggered by female-specific chemosensory signals (other than sex pheromone) primarily detected by antennae. Promotion of ootheca production between females is likely to be an early stage of social cooperation, reminiscent of the foundation and maintenance of a colony by female pairs in the eusocial termite *Reticulitermes speratus*.

**Electronic supplementary material:**

The online version of this article (doi:10.1186/s40851-017-0063-x) contains supplementary material, which is available to authorized users.

## Background

Parthenogenesis is a mode of asexual reproduction in which offspring are produced by females without the genetic contribution of a male. This occurs in many animal phyla, from rotifiers, nematodes and arthropods to some lower vertebrates [1–4]. Parthenogenesis results in lower fitness in the long term, because offspring do not generate much genetic diversity [1]. However, in the short term, especially in the presence of abundant resources, parthenogenesis can be a useful strategy for rapidly generating large numbers of female progeny and colonize new habitats, as is known to occur in aphids [1, 4].

In most animal groups, parthenogenesis is a strategy secondary to sexual reproduction and occurs only when mating partners (males) are unavailable [1]. Developmental constraints of parthenogens often prevent the evolution of parthenogenesis from a sexually reproducing species [4, 5]. Since most animals that show obligatory parthenogenesis occupy the terminal nodes of phylogenetic trees, the evolutional origin of parthenogenesis could be attributed to the acquisition of a switching mechanism from sexual reproduction to facultative parthenogenesis in more basal taxa [1].

In this context, Blattodea (cockroaches and termites) represent an intriguing phylogenetic group from which sexual reproduction, facultative parthenogenesis and more obligatory parthenogenesis have diversely emerged [4, 6]. This specific form of parthenogenesis is known as “thelytoky,” in which females produce only females from unfertilized eggs [4]. For example, the speckled cockroach *Nauphoeta cineria* reproduces by facultative parthenogenesis; that is, some are capable of switching from a sexual mode of reproduction to an asexual mode when isolated from males [7, 8]. However, the fitness of parthenogenetically reproducing females is significantly lower than that of sexually reproducing females [8]. In contrast, the Surinam cockroach *Pycnoscelus surinamensis* exhibits obligatory parthenogenesis; individuals endemic to Indo-Malaysian regions reproduce sexually, but those that were accidentally introduced by humans to other areas such as the USA and Australia reproduce only asexually [9, 10]. Geographic parthenogenesis is also known in *Phyllodromica subaptera,* in which asexual forms have spread through most Mediterranean countries, while sexual forms are found only on the Iberian Peninsula [11]. In eusocial termites, *Reticulitermes speratus*, a female-female colony is formed when kings are not available and is maintained by parthenogenesis [12–14]. Similarly, queen succession in the presence of a king is also maintained by automictic parthenogenesis with terminal fusion [15].

The American cockroach, *Periplaneta americana* (L.) (Insecta: Blattodea: Blattoidea: Blattidae) is a worldwide pest due to its euryphagous, gregarious behavioral ecologies and close association with human habitats [16]. This species is phylogenetically closer to termites (Blattodea: Blattoidea) than the members of the suborder Blaberoidea, which includes *N. cineria*, *P. surinamensis* and the German cockroach *Blattera germanica* [17, 18]. Females of *P. americana* show facultative parthenogenesis in the absence of males [19, 20]. Although the hatchability of eggs produced by parthenogenesis is lower than that of eggs produced by sexual reproduction [20], the resultant female offspring have been shown to survive through at least two generations in the laboratory [19].

It could be speculated that the ability of females to sense “male-absent conditions” is important in triggering parthenogenesis. However, the decision-making process is not straightforward in group-living animals. Females must be able not only to discriminate other individuals based on sex and kinship [21] but also to evaluate the density and reproductive quality of individuals in populations [5]. There has been no systematic study on how population density affects asexual reproduction in group-living animals (i.e., group effect).

The aim of the present study was to clarify the effect of grouping on asexual ootheca production and to gain insight into sensory cues underlying the promotion of parthenogenesis. Using behavioral assays, we investigated the effects of grouping on asexual ootheca production. Since oothecae delivered to the abdominal tip are soon deposited in *P. americana*, the precise timing of ootheca production can easily be determined by checking the abdominal tip. Our results show that grouping of females indeed promotes ootheca production, suggesting that this is an early stage of social cooperation, preadaptive to more prevalent parthenogenesis.

## Methods

### Insects

Adult virgin cockroaches (*Periplaneta americana*), reared in a 12:12 h light-dark cycle at 28 °C, were used in this study. Laboratory colonies including nymphs and adults of different ages were maintained for approximately four years, during which time wild individuals were added *ad libitum* to prevent inbreeding. Both males and females were kept separated in the stage of final larval instar to prevent mating and contamination of sex-specific odor. Immediately after the imaginal molt, individuals were used for behavioral observations. Unless otherwise stated, adults with intact genitalia and olfactory organs (pairs of antennae, maxillary palps, and labial palps) were used.

### Behavioral assay

**Fig. 1:**
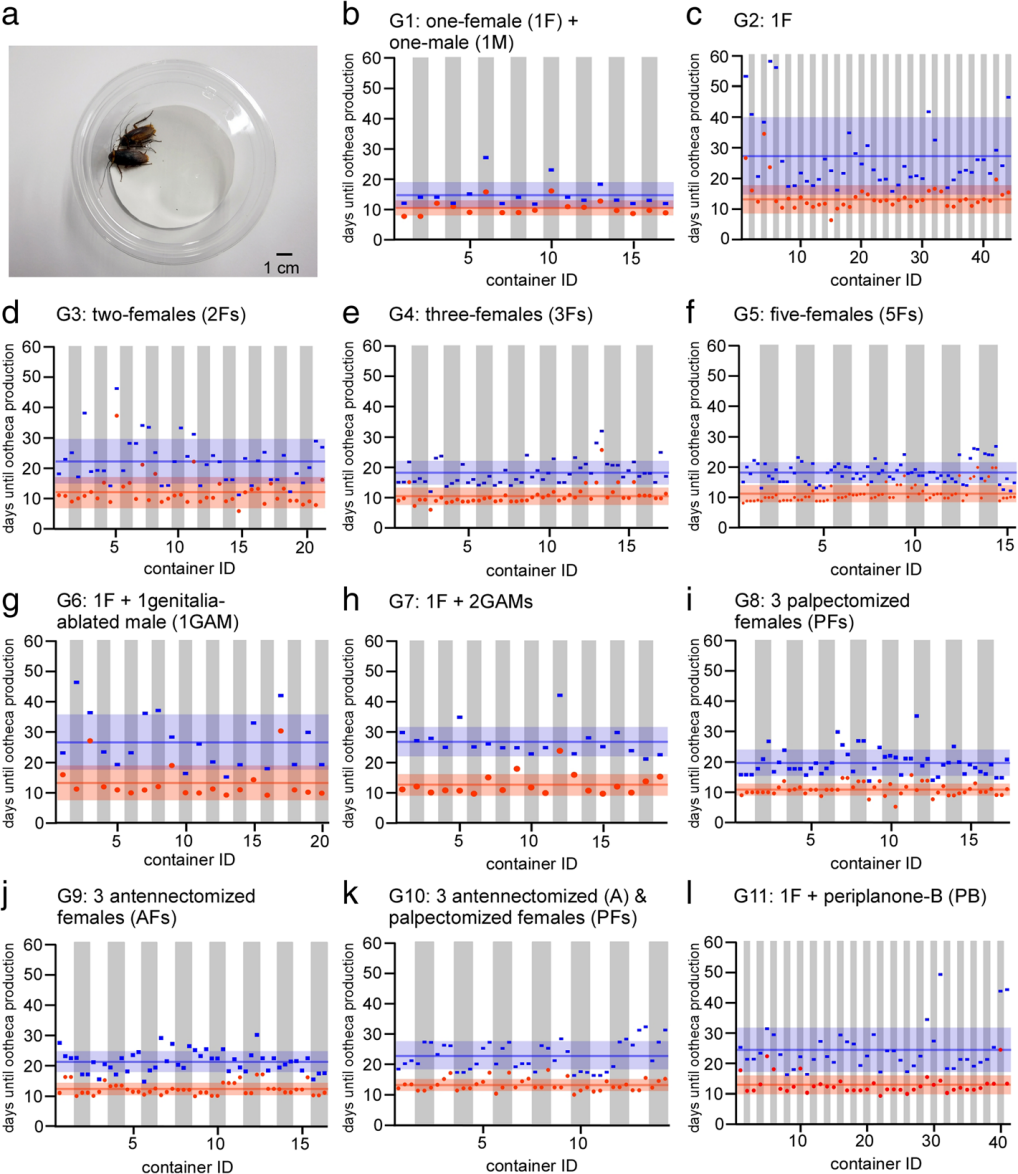
Timing of the first and second ootheca productions in 11 experimental groups (G1-G11). **a** Photograph of two female cockroaches housed in a circular plastic container for the behavioral assay. **b**-**l** Scatter plots showing intervals between the imaginal molt and the first ootheca production (*red*) and the second ootheca production (*blue*) of individual females in different experimental groups. Each dot represents the interval of identified individuals. *Red* and *blue* lines indicate the mean period for the first and the second ootheca productions and pale *red* and *blue* bands indicate the width of the standard deviation for the first and the second ootheca productions

During observation, a cockroach or cockroaches were kept in a sealed circular plastic container (diameter = 11 cm; height = 6 cm; volume = 450 cm^3^, see Fig. 1a). The bottom of the container was lined with a filter paper (Fig. 1a). Minimum aeration was assured by making two small ventilation holes (ø 2 mm) in the lid. The cockroaches were fed insect food pellets (Oriental Yeast, CO., LTD, Japan) and carrots and given water *ad libitum*. Individual cockroaches in a single container were marked by trimming forewings to different shapes. Ootheca production was checked visually when a new ootheca was delivered to the tip of the abdomen. In our experimental conditions, we did not observe empty oothecae or resorption of oocytes. We recorded the interval between the imaginal molt and the first ootheca production, and that between the imaginal molt and the second ootheca production.

We conducted 11 sets of behavioral experiments during the same period, from August to 2013 to January 2014 (Table 1). As a control, one female was kept together with one male, permitting sexual reproduction (group 1). Secondly, to evaluate the group-housed effect on asexual reproduction, one female (group 2) was kept in a container and two (group 3), three (group 4) and five females (group 5) were kept together in the container. Thirdly, to assess effects of the presence of males on asexual reproduction, one female was kept with one genitalia-ablated male (group 6) or two genitalia-ablated males (group 7). Fourthly, to evaluate possible sensory organs influencing asexual reproduction, three females underwent ablation of maxillary/labial palps (group 8), pairs of antennae (group 9), and all antennae/palps (group 10). Finally, to evaluate the effect of sex pheromones on asexual reproduction, a primary component of sex pheromones, periplanone-B (PB: originally synthesized by Drs. S. Kuwahara and K. Mori and kindly donated by Dr. M. Willis at Case Western Reserve University) [22] was added to a container housing one female (group 11). Periplanone-B is known to attract distant males and elicit the complete sequence of the male mating display [23].

**Table 1:** Intervals to the first and second ootheca productions from the imaginal molt in experimental groups

Group	Reproduction mode	1st ootheca production (mean ± SD)	CV	2nd ootheca production (mean ± SD)	CV	Number
One female with one male	sexual	10.5 (±2.5)	0.24	14.7 (±4.3)	0.29	17
One female	asexual	13.2 (±4.7)	0.36	27.0 (±12.9)	0.48	44
Two females	asexual	11.9 (±5.2)	0.44	22.0 (±7.5)	0.34	42
Three females	asexual	10.4 (±2.9)	0.28	18.2 (±4.0)	0.22	51
Five females	asexual	11.1 (±2.9)	0.26	18.1 (±3.5)	0.19	75
One female with one genitalia ablated male	asexual	13.0 (±5.8)	0.44	26.3 (±9.1)	0.35	20
One female with two genitalia ablated males	asexual	12.6 (±3.6)	0.28	26.6 (±4.9)	0.18	19
Three palpectomized females	asexual	10.9 (±2.0)	0.19	19.6 (±4.3)	0.22	51
Three antennectomized females	asexual	11.9 (±2.0)	0.17	20.8 (±3.5)	0.17	48
Three antennae and palpectomised females	asexual	12.8 (±2.0)	0.15	22.4 (±4.7)	0.21	42
One female with periplanone-B	asexual	12.6 (±3.1)	0.25	24.1 (±7.4)	0.31	41

*CV* coefficient of variation

For surgical ablation of sensory organs, cockroaches were briefly anesthetized by carbon dioxide. Pairs of the entire antennal flagella and palps were excised at their base using microscissors (Vananas Scissors, 500086, WPI). Removal of antennae or palps had little effect on feeding behavior. However, females without antennae and palps tended to be inactive for approximately three days before starting to feed. In males, all hooks of the phallomeres of genitalia [24], indispensable for holding the female genitalia in mating, were removed using microscissors (Vananas Scissors, 501778, WPI). We confirmed that the operated males fail to copulate permanently, but other behaviors, such as excretion and courtship behaviors, were unaffected. For sex pheromone application, 1.0 ng synthetic (−) PB, somewhat larger than the daily release of PB (0.6 ng) by one virgin female [25], was dissolved in 10 μl n-hexane, and a strip of filter paper (3 × 10 mm) was immersed in the solution. Immediately after n-hexane had evaporated, the strip was placed on the bottom of a container at the beginning of scotophase. The strip was replaced with a new one each day.

### Viability check of asexually and sexually produced oothecae

To evaluate the viability of eggs produced by parthenogenesis and normal sexual reproduction in our laboratory colony, we collected 33 deposited oothecae from a colony of 20 virgin females and 30 deposited oothecae from a colony containing 10 virgin females and 10 virgin males that were allowed to mate freely. Each ootheca was housed in a 1.5 ml Eppendorf® Safe-lock tube with two small holes (0.5 mm) in the lid for aeration and incubated at 28 °C. The number of eggs hatched from each ootheca was checked visually over a period of two months until all offspring were confirmed to have hatched.

### Statistical analysis

Unless otherwise stated, all values in Results are represented as means ± SD. The sample size in each group is shown in Table 1. To indicate the extent of variability in relation to the mean of the population, the coefficient of variation (CV) was defined as the ratio of standard deviation to the mean (Table 1). To compare significance of the mean value, analysis of variance (ANOVA) was conducted for all 11 groups. Subsequently, multiple comparisons were made by the Games-Howell test using add-ins attached to Excel (Excel statistics ver. 7.0, Esumi, Japan). Welch’s *t*-test was conducted to evaluate hatchability of oothecae, their lengths, and mean number of nymphs per ootheca in sexual and asexual reproductions (Table 2).

**Table 2:** Viability of sexually and asexually produced oothecae

	Total oothecae	Hatched oothecae	Hatchability (%)^a^	lengths of hatched ootheca (mm)^b^	Lengths of unhatched ootheca (mm)^c^	Mean no. of nymphs per ootheca (±SD)^d^
Mated	30	14	46.7	8.2 ± 0.8	8.4 ± 0.6	14.3(±1.5)
Unmated	33	10	30.3	8.7 ± 0.7	8.7 ± 0.5	9.1 (±3.3)*

Oothecae produced by unmated females were collected from a colony of twenty virgin females. Oothecae were incubated at 28 °C in a 12:12 h light-dark cycle. Welch’s *t*-test *P* = 0.18 (>0.05)^a^, 0.098 (>0.05)^b^, 0.156 (>0.05)^c^, 0.00004 (<0.0001)^d^. Asterisk indicates the presence of statistical significance

## Results

### Ootheca production in isolated females (parthenogenesis) and paired female-male (sexual reproduction)

**Fig. 2:**
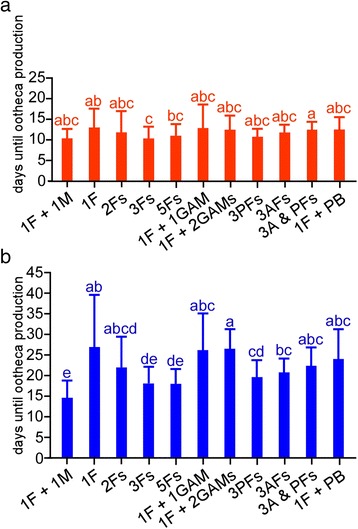
Statistical evaluation of different experimental groups. **a**, **b** Bar graphs of mean intervals between the imaginal molt and the first ootheca production (**a**, *red*) and the second ootheca production (**b**, *blue*) in experimental groups (see Fig. 1 for full spellings of the abbreviated terms for experimental groups). Error bar represents the standard deviation (SD) of the mean. ANOVA (**a**): F_10, 439_ = 3.744, *P* = 7.6 × 10^−5^. ANOVA (**b**): F_10, 439_ = 11.291, *P* = 2.9 × 10^−17^. Means not sharing the same letter are significantly different (Games-Howell, *P* < 0.05)

The virgin females isolated immediately after the imaginal molt produced the first ootheca via parthenogenesis at 13.2 ± 4.7 days (Figs. 1c and 2a, Table 1), which was not significantly different from that in mated females (10.5 ± 2.5 days, Figs. 1b and 2a, Table 1). However, the second ootheca production was significantly delayed with more variance among individuals (27.0 ± 12.9 days, CV = 0.48, Figs. 1c and 2b, Table 1) compared to that in mated females (14.7 ± 4.3 days, CV = 0.29, Figs. 1b and 2b). These results are generally in good agreement with the results of a previous study [20].

### Ootheca production in group-housed females

When multiple females were kept in the same container, the ootheca production cycle tended to be shortened, with less variance among individuals compared to that in isolated females (Fig. 1d-f, see CVs in Table 1). Apparent synchronization of ootheca production occurred not only in females housed in the same container, but also those housed in different containers (Fig. 1e, f); thus, this is a substantially different phenomenon from menstrual synchrony reported in women living in close proximity [26].

The shortening and synchronization of the ootheca production cycle became more prominent as the number of housed females increased (Fig. 1d-f, Table 1). For example, the duration until the first ootheca production in three females was significantly shorter than that in isolated females (Fig. 2a, Table 1; 10.4 ± 2.9 days for the first ootheca production in three females). The durations until the second ootheca production in three females and five females were significantly shorter than that in isolated females (Fig. 2b, Table 1; 18.2±4.0 for the second ootheca production in three females; 18.1 ± 3.5 days for the second ootheca production in five females, Fig. 1f).

### Effect of sexual difference of cohabitants on ootheca formation

To determine the effect of sexual difference of the cohabitants on ootheca production by females, we compared the results when a virgin female was housed with genitalia-ablated males (Fig. 1g, h) to the results when a female was housed with other virgin females (Fig. 1d, e).

When virgin females were paired with genitalia-ablated males, the first and second ootheca productions (Fig. 1g, h) were not significantly different from those in isolated females (Fig. 1c), but were delayed compared to those in female-only groups (Fig. 1d, e, Table 1). When a single female was kept with one genitalia-ablated male, the female produced the first ootheca and the second ootheca at 13.0 ± 5.8 days and 26.3 ± 9.1 days (Figs. 1g and 2, Table 1), respectively, which were delayed, but not significantly compared to cases in which a single female was kept with another female (Figs. 1d and 2, 11.9 ± 5.2 days for first ootheca production and 22.0 ± 7.5 days for second ootheca production). However, when a single female was kept with two genitalia-ablated males, the female produced the second oothecae significantly later (Figs. 1h and 2b, 26.6 ± 4.9 days) than that when a female was kept with two females (Fig. 1e, 18.2 ± 4.0 days).

### Sensory organs for discriminating sex of cohabitants

We evaluated the effect of ablation of primary chemosensory organs (antennae and palps) [16] on ootheca production, because chemosensory signals are known to be important for discrimination of the sex of cohabitants in other cockroach species [27].

Removal of the maxillary/labial palps had little effect on ootheca production in females; the durations to the first and second ootheca production in three females (Figs. 1i and 2a, b, Table 1, 10.9 ± 2.0 and 19.6 ± 4.3 days, respectively) were not significantly different from those in intact females (Figs. 1e and 2a, b). Antennal removal resulted in a significant delay in the second ootheca productions (Figs. 1j and 2b, Table 1, 20.8 ± 3.5 days) compared to that in intact females (Table 1, Figs. 1e and 2b), suggesting that antennae play a more important role than palps in shortening the duration to ootheca production. Moreover, removal of both antennae and maxillary/labial palps resulted in even greater delays in the first ootheca production (Figs. 1k and 2a, Table 1, 12.8 ± 2.0 days) and second ootheca production (Figs. 1k and 2b, Table 1, 22.4 ± 4.7 days).

### Effect of a sex pheromone on ootheca formation

Since sex pheromones are emitted by virgin females but much less by mated females in *P. americana* [25], they may be chemicals that signal a male-absent condition. We therefore investigated whether ootheca production is promoted by daily addition of 1.0 ng PB to the container housing single females at the beginning of scotophase. However, the results were negative; the durations to first and second ootheca production were not significantly shortened in isolated females exposed to PB compared to those in isolated females without PB (Fig. 1l, Table 1).

### Notes on parthenogenesis in *P. americana*

**Fig. 3:**
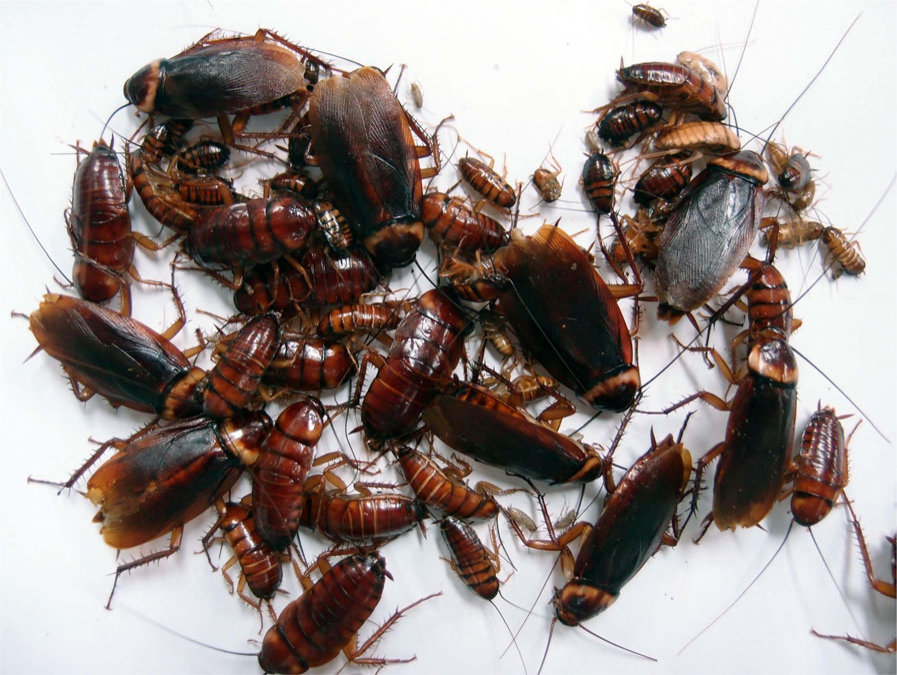
Photograph of offspring collected from a colony maintained by parthenogenesis. The offspring, anesthetized briefly by carbon dioxide, are all females and had reached the fifth generation since a colony had been founded by 15 virgin females more than three year ago. No malformed adults or nymphs were detected

Finally, we evaluated how developmental viability differs in sexual reproduction and asexual reproduction. Surprisingly, we found that parthenogenesis in *P. americana* is more sustainable than previously thought [19, 20]. We prepared a founder colony of 15 virgin adult females randomly collected from the laboratory colony in December 2013. More than 300 females with different-aged nymphs and adults have survived as of February 2017. Several individuals were photographed immediately after brief anesthetization (Fig. 3). Since the colony has been given food and water *ad libitum* and kept at an appropriate temperature (28 °C), some individuals may have reached the fifth generation.

The hatchability of oothecae produced by a colony of 20 virgin females was 30.3% (Table 2), lower than that of oothecae produced by sexual reproduction (46.7%, Table 2). The lengths of oothecae produced by asexual reproduction and sexual reproduction were similar, regardless of whether they were hatched or not (Table 2, Additional file 1). The mean numbers of nymphs hatched from each ootheca were significantly smaller for parthenogenesis (9.1 ± 3.3) than those in sexual reproduction (14.3 ± 1.5, Table 2; Additional file 1). These results suggest that parthenogenetic eggs show lower fitness than sexually produced eggs, but the difference is smaller than that in the results of a previous study in which the hatchability of parthenogenetic oothecae was 37.0%, and that of oothecae produced by sexual reproduction was 60.0% with mean numbers of nymphs hatched from each ootheca being 4.9 for parthenogenesis and 10.8 for sexual reproduction [19].

## Discussion

In group-living animals, grouping of conspecifics decreases mortality rate [28] and promotes nymphal development [28–31], egg production [32], and development of the related endocrine system [32, 33]. These phenomena have been referred to as the “group effect” or “effect of population density”.

Our study showed that a group effect is exerted not only on the reproductive mode of females, but also on the parthenogenetic mode of females. In the presence of sufficient resources, the group-housed females after the imaginal molt tended to produce parthenogenetic oothecae earlier and at more similar timings than did isolated females. Since shortening and synchronization of ootheca production cycle occurred not only in those housed in the same container, but also in those kept in different containers, the overall effect of promotion of ootheca production appears to reach a plateau for each individual, resulting in apparent synchronization of ootheca production. This assumption is supported by the finding that the shortening of the ootheca production cycle when five females were grouped was similar to that when three females were grouped. The cycle of parthenogenetic ootheca production is the fastest ever known in this species [19, 20, 33].

Ootheca production was significantly promoted when females were housed with virgin females but not when they were housed with genitalia-ablated males, suggesting that discrimination of cohabitants’ sex is a prerequisite for females to promote ootheca production. Ablation of the largest chemosensory organs, antennae, resulted in a delay in ootheca production compared to that in intact females, although complete ablation of the antennae may have side-effects related to loss of sensory input [34]. Unexpectedly, a typical female-specific odor, the primary sex pheromone component (periplanone-B), did not promote ootheca production, despite the fact that the female *P. americana* is equipped with a specific olfactory glomerulus in its first-order olfactory center that processes periplanone-B [35, 36].

Given that chemosensory signals are utilized for fine discrimination of the cohabitant status in cockroaches [21, 27], we suspect that sensing female-specific odors other than sex pheromones and/or sensing nonvolatile chemicals (e.g., hydrocarbons) [37] via antennal contact with females is most potent for promoting ootheca production. However, our data do not negate the possibility that mechanosensation is involved in the group effect. Although the values were below the significance threshold, some degree of shortening and synchronization of oothecae production occurred in females even without chemosensory organs. Given that tactile stimulation promotes ovary maturation in *B. germanica* [34, 38], tactile inputs from mechanosensory bristles distributed throughout the body and legs may complementarily promote asexual ootheca production.

On the level of endocrine control, juvenile hormone (JH) III released by the *corpus allatum* has a pivotal role in promoting the rate of vitellogenic growth and subsequent ootheca production in females [39]. In *B. germanica*, isolated virgin females have significantly lower rates of JH III synthesis than those in grouped females [40]. Further study is needed to evaluate whether JH III synthesis in *P. americana* is promoted more in group-housed females than in isolated females.

What is the functional significance of promotion of asexual ootheca production between females? Shortening of the ootheca production cycle contributes to an increase in parthenogenetic offspring produced by one female. Moreover, synchronizing egg production in grouped females may result in similar hatching timing of their offspring. Nymphs hatched synchronously would increase their fitness by aggregation and by sharing of resources, which could counter the lower hatching rate of parthenogenetic eggs than that of sexually produced eggs [19, 20].

In pre-social, domiciliary cockroaches, females of the same kin tend to aggregate in the same colony, whereas males leave the colony to avoid inbreeding [41]. Our behavioral observations are consistent with this finding; unmated females housed in the same container huddle close together with almost no fighting, whereas paired unmated males often fight until the antennae of both individuals are amputated (Nishino, personal observation). Thus, recognition of other virgin females and subsequent promotion of ootheca production might be the early stage of social cooperation that drives more prevalent parthenogenesis. This cooperative behavior is possibly succeeded by eusocial termites, five *Reticulitermes* species that found the first colony by female-female cooperation [13, 42].

As exemplified by *P. surinamensis and P. subaptera*, obligatory parthenogenesis very likely arises from facultative parthenogenesis in areas with low population densities. Females are advantageous over males for survival with low population densities. For example, females of *P. americana*, especially unmated ones, live longer than males [19, 43]. Due to their larger body masses, females are resistant to environmental changes, such as desiccation [9]. Thus, these traits of females appear to be suitable for adapting to new habitats with unfavorable conditions and maintaining female populations via parthenogenesis.

Maintaining certain populations of *Periplaneta* for more than four generations over a period of three years only by parthenogenesis is a threat to public health because of their potential roles as vectors for pathogens [44, 45] and allergens [46] indoors.

The fitness of parthenogens of *P. americana* is estimated to be higher than that of another species that uses facultative parthenogenesis, *N. cineria*, in which the clutch size of asexually produced offspring (3.2 ± 2.4) is much lower than that of sexually produced offspring (23.6 ± 4.2) and parthenogized progeny do not survive beyond the third generation [8]. Therefore, care should be taken for the possibility that a female-only colony of *P. americana* may be maintained locally, since benign but spatially isolated conditions can be created in sewage systems in most urban cities. Further investigation is clearly needed to determine whether the sustainability of a female-only colony is due to the inherent nature of wild individuals or to the genetic shift through artificial selection in our laboratory colonies.

One important but yet unsolved issue is whether the automictic parthenogenesis (meiosis and subsequent restoration by the doubling of chromosomes) opted by termites [13] is the case for more basal Blattoidea, *P. american*a. Microsatellite genotyping is needed to understand what kind of reproductive mode contributes to the maintenance of genotypic variance to counter the lower fitness of thelytoky parthenogenesis, and this is probably the key for a deeper understanding of why the genus *Periplaneta* is so abundant worldwide.

## Conclusions

Group-housed females of the American cockroach *P. americana* promote asexual ootheca production compared to isolated females. A founder colony of 15 virgin females is sufficient to maintain the colony for more than four generations over a period of more than three years only by parthenogenesis. Recognizing female-specific chemosensory signals via antennae is the most potent sensory cue for promoting ootheca production. The promotion of ootheca production may be an early stage of social cooperation linked to more prevalent parthenogenesis adopted by eusocial insects.

## Additional file

Additional file 1: Raw data shown in Table 2. Raw data about lengths of hatched oothecae, lengths of unhatched oothecae and mean numbers of nymphs per hatched oothecae are shown under the Table. (XLSX 11 kb)

## Abbreviations

CV: Coefficient of variation
JH: Juvenile hormone
PB: Periplanone-B
